# Klinefelter syndrome (KS): genetics, clinical phenotype and hypogonadism

**DOI:** 10.1007/s40618-016-0541-6

**Published:** 2016-09-19

**Authors:** M. Bonomi, V. Rochira, D. Pasquali, G. Balercia, E. A. Jannini, A. Ferlin, On behalf of the Klinefelter ItaliaN Group (KING), Giancarlo Balercia, Marco Bonomi, Aldo Calogero, Giovanni Corona, Andrea Fabbri, Alberto Ferlin, Felice Francavilla, Vito Giagulli, Fabio Lanfranco, Mario Maggi, Daniela Pasquali, Rosario Pivonello, Alessandro Pizzocaro, Rosario Pivonello, Antonio Radicioni, Vincenzo Rochira, Linda Accardo, Biagio Cangiano, Rosita A. Condorelli, Giuliana Cordeschi, Settimio D’Andrea, Antonella Di Mambro, Daniela Esposito, Carlo Foresta, Sandro Francavilla, Mariano Galdiero, Andrea Garolla, Lara Giovannini, Antonio R. M. Balercia, Sandro La Vignera, Giovanna Motta, Luciano Luciano, Fiore Pelliccione, Luca Persani, Daniele Santi, Riccardo Selice, Manuela Simoni, Carla Tatone, Giacomo Tirabassi, Alberto Stefano Tresoldi, Enzo Vicari

**Affiliations:** 1 Department of Clinical Sciences and Community Health, University of Milan, Milan, Italy; 2 Division of Endocrine and Metabolic Diseases & Laboratory of Endocrine and Metabolic Research, IRCCS Istituto Auxologico Italiano, Milan, Italy; 3 Unit of Endocrinology, Department of Biomedical, Metabolic and Neural Sciences, University of Modena and Reggio Emilia, Via P. Giardini 1355, 41126 Modena, Italy; 4 Azienda USL of Modena, NOCSAE, Via P. Giardini 1355, 41126 Modena, Italy; 5 Department of Cardiothoracic and Respiratory Science, Second University of Naples, Naples, Italy; 6 Division of Endocrinology, Department of Clinical and Molecular Sciences, Umberto I Hospital, Polytechnic University of Marche, Via Conca 71, 60126 Ancona, Italy; 7 Department of Systems Medicine, Tor Vergata University of Rome, Rome, Italy; 8 Unit of Andrology and Reproductive Medicine, Department of Medicine, University of Padova, Padova, Italy

**Keywords:** Klinefelter syndrome, KS, Testosterone, Hypergonadotropic hypogonadism, Chromosome abnormalities, Azoospermia, Male infertility

## Abstract

Klinefelter Syndrome (KS) is characterized by an extreme heterogeneity in its clinical and genetic presentation. The relationship between clinical phenotype and genetic background has been partially disclosed; nevertheless, physicians are aware that several aspects concerning this issue are far to be fully understood. By improving our knowledge on the role of some genetic aspects as well as on the KS, patients’ interindividual differences in terms of health status will result in a better management of this chromosomal disease. The aim of this review is to provide an update on both genetic and clinical phenotype and their interrelationships.

## Introduction

In 1942, Klinefelter et al. [1] published a report on 9 men who had enlarged breasts, sparse facial and body hair, small testes, and an inability to produce sperm. In 1959, these men with Klinefelter syndrome (KS) were discovered to have an extra X chromosome (genotype XXY) instead of the usual male sex complement (genotype XY). The classic form of KS, which is present in the 80–90 % of the cases, is defined by a 47,XXY karyotype resulting from the aneuploidy of the sex chromosomes, whereas higher-grade aneuploidies (e.g. 48,XXXY or 48,XXYY), structurally abnormal X chromosome (e.g. 47,iXq,Y) or mosaicisms (e.g. 47,XXY/46,XY) make up approximately in the remaining 10–20 % of cases. The prevalence of KS (ranging from 0.1 to 0.2 % in newborn male infants) rises up to 3–4 % among infertile males and 10–12 % in azoospermic patients [2], and it is the most frequent observed sex chromosomal anomaly, with an estimated frequency of 1:500 to 1:1000 men [3]. KS has increased in the last years [4] although in the absence of a concomitant rise in the prevalence of XXY aneuploidy. This may indicate that the rise of the KS might be related to the increasing of the paternal meiotic alterations. KS patients have a phenotype which is extremely variable, but without any obvious facial dysmorphology that make them indistinguishable from the boys with normal karyotype [5].

KS is associated with several clinical conditions coming from both the genetic abnormalities and hypogonadism. The aim of this review is to discuss KS clinical features according with the genetic and hormonal (low testosterone) factors involved in their pathogenesis.

## Clinical phenotype and hypogonadism

### Clinical phenotype

**Fig. 1:**
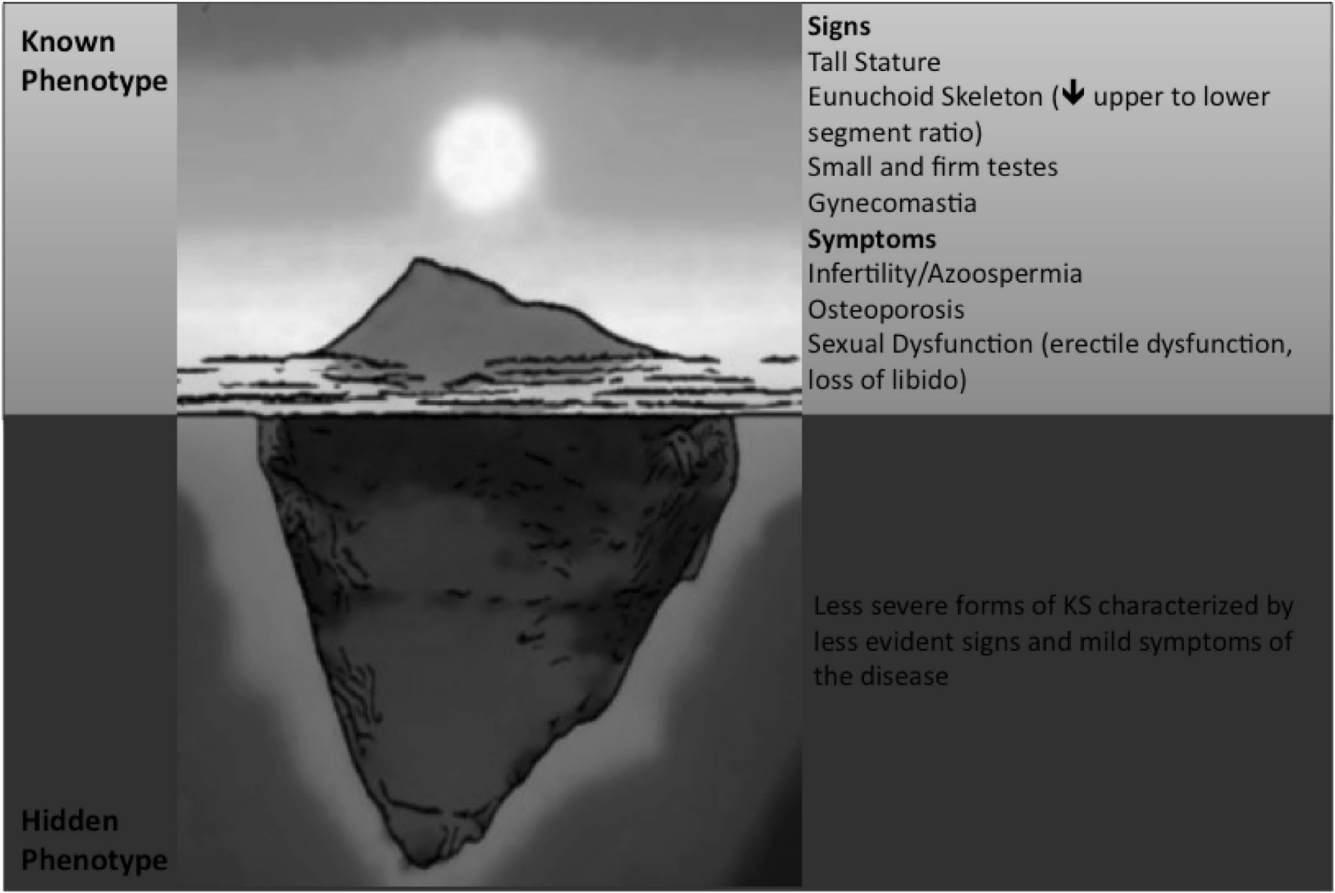
Signs and symptoms of KS according to the severity of clinical phenotype

The major signs and symptoms of Klinefelter Syndrome (KS) have been well characterized since the first description of the disease [1]. As traditionally described, patients with KS have tall stature, small testes, gynecomastia in late puberty, gynoid aspect of hips (broad hips), sparse body hair, signs of androgen deficiency and low serum testosterone coupled with elevated gonadotropins, and finally azoospermia, oligospermia with hyalinization and fibrosis of the seminiferous tubules [5, 6] (Fig. 1). Usually, the above-mentioned signs of hypogonadism are also coupled with psychosocial problems, although an alternative phenotype has also been described, characterized by fewer clinical features.

Clinical features depend on both the supernumerary X chromosome and the effects of hypogonadism [7]. However, what we know about signs and symptoms of KS renders about the tip of the iceberg (Fig. 1) since most of the patients with KS remain overlooked [5]. It has been estimated that the prevalence of the KS is greater than the number of patients who really had received a clinical diagnosis thanks to the comparison of epidemiological data coming from prenatal diagnosis with those obtained from men who have been diagnosed after birth [3, 8]. Unfortunately, the clinical picture of men with KS in the form we know (as settled by data available in the literature) comes directly from the description of men who had received a certain diagnosis of KS [5, 8]. Hence, signs and symptoms at the base of the iceberg of the classical clinical phenotype of KS remain still to be completely unraveled (Fig. 1). Indeed, the classical phenotype described above has been characterized only on the basis of a small number of affected patients, precisely those seeking medical consultation and probably displaying the most severe degree of clinical features.

#### The broad spectrum of phenotypes in KS

**Fig. 2:**
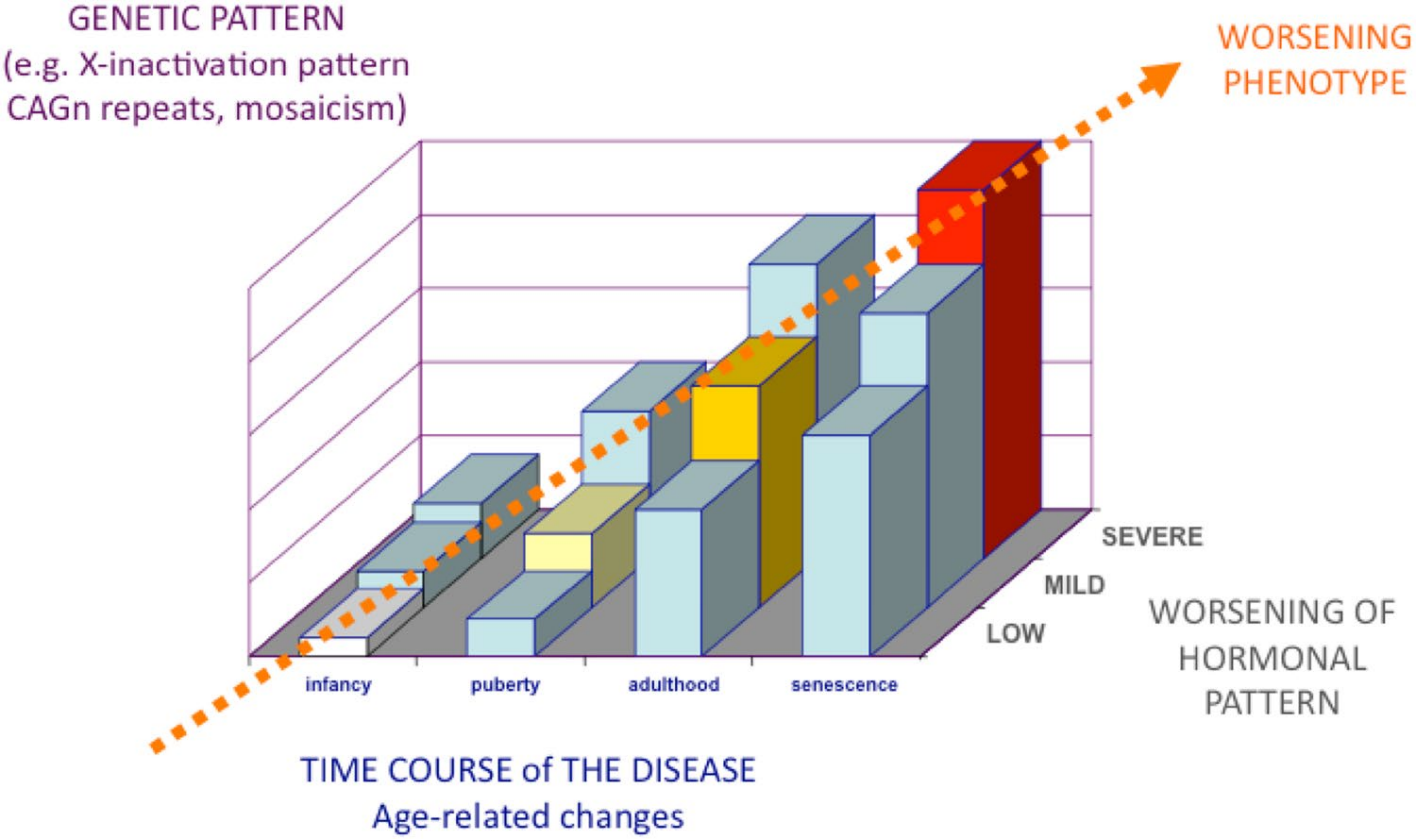
The broad spectrum of phenotypes in KS depends on the severity of all its components (number of supernumerary X chromosome, genetic impact of supernumerary X, severity of hypogonadism) as well as on the time duration of the disease, the delay in the diagnosis of testosterone deficiency, and advancing age coupled with increasing other comorbidities

In spite of the clinical phenotype of men with KS, as classically described in the literature, a parallel, less described phenotype has also been recognized, in which patients present with fewer clinical features [9]. These less severe or mild forms (most of which remain often undiagnosed) are characterized by paucisymptomatic manifestations [5, 10, 11]. Thus, the real complete spectrum of different KS phenotypes remains still to be fully elucidated in detail (Fig. 1). Probably, the phenotype depends on the severity of the expression of genetic defect, androgen deficiency, and androgen receptor sensitivity (i.e., CAG repeats polymorphism) [12]. The more the genetic expression, androgen deficiency, and androgen receptor sensitivity are worse, the more the phenotype will be severe [13] (Fig. 2). Less severe forms of genetic abnormalities, such as mosaicism, generally result in both less severe clinical symptoms and endocrine abnormalities [14], while the phenotype progressively worsens with the severity of polysomy (e.g. 49, XXXXY) [5, 7, 10, 11]. Language and speech disabilities increases with the increase of supernumerary X chromosome and seem to contribute decreasing of 15–16 points of intelligence quotient (IQ) per each extra X chromosome [15].

The high frequency of mild phenotypes explains, at least in part, why most of the patients with KS remain undiagnosed [6] and claims for efforts in improving our ability to promptly reach a diagnosis. Since symptoms rarely present simultaneously, the disease remains often overlooked and the diagnosis is missed or delayed. It has been estimated that many cases remain undiagnosed and only 26 % of the expected number of KS adults are correctly identified late in adult life, leading to severe complications and a more difficult clinical management [16].

#### Relationship between age and KS phenotype

Signs and symptoms appearance depend also on patients’ age. Furthermore, the phenotype tends to worsen with advancing age (Fig. 2), according to the increasing number of features and comorbidities that accumulate with aging and to the exacerbation of those already present. The time of onset of clinical signs and symptoms depends on patient’s age in men with KS. The timing of the clinical features appearance allows identifying both androgen-dependent and supernumerary X-dependent signs and symptoms (Table 1). Distinguishing symptoms related to androgen deficiency from that due to chromosomal abnormalities is crucial in order to improve the outcome of testosterone replacement therapy, to establish how the disease should be monitored during the follow-up, and to inform the patient on what the expected results are [17]. Signs and symptoms appearing during infancy such as longer legs [17, 18] and speech disabilities [19, 20] have been attributed to the genetic abnormality rather than to hypogonadism [5, 7, 10] (Table 1). Even though rare in KS [7, 21], genital anomalies (micropenis, undescended testis, bifid scrotum and hypospadia) might present at birth, but if they are due to the effects of supernumerary X chromosome/s or of androgen deficiency during fetal life remains still to be determined [5] (Table 1).

**Table 1:** Phenotypic features of KS grouped according to the underlying pathogenetic mechanism

	Features due to supernumerary X chromosome	Features due to Testosterone deficiency	Features due to both supernumerary X chromosome and Testosterone deficiency
Onset Time	Before Puberty	At puberty or during adulthood	Before puberty with progressive worsening after puberty
Signs	Congenital malformations (cleft palate, hernia) [rare] Longer legs Small testes	Sparse body and facial hair Female pubic escutcheon Reduced muscle mass Bilateral gynecomastia Eunuchoid skeleton Impaired estradiol/testosterone ratio Longer legs? [T-deficiency during fetal life]??	Eunuchoid skeletal proportions Gynoid hips Tall stature Genital abnormalities at birth [rare] Elevated gonadotropins BMI in the range of overweight or obesity Metabolic abnormalities Reduced bone mineral density
Symptoms	Speech and language disabilities Azoospermia	Impaired sexual desire Impaired erectile function Weakness and loss of vigor Impaired well-being	Mood disturbances

The main sign, which is always present in KS, is represented by small testes. At puberty, both the sexual development and the growth spurt generally proceed in a normal way, but the progressive increase in testes volume does not occur, both testes remaining small (<4 mL in volume) and firm [1, 5, 7, 10, 11]. Thus, testes volume does not increase at puberty while the penis and secondary sexual characteristics progress in a normal fashion through all the pubertal stages.

The degree of virilization varies widely in adult men with KS, but it shows a tendency to decrease and to worsen progressively with advancing age (Table 1), similarly to what happens to other clinical conditions associated with KS such as diabetes and metabolic syndrome [16, 22, 23]. Accordingly, after the age of 25, about 80 % of men with KS complain of symptoms related to overt hypogonadism (decreased libido, erectile dysfunction) [5, 24].

## Genetics and clinical phenotype

### The genetic phenotype

The genetic background for the KS is based on sex chromosome non-disjunction, which leads to the presence of extra X chromosome/s. Indeed, non-disjunction represent the failure of chromosome to separate at anaphase during meiosis I, meiosis II or mitosis giving rise to cells with an aberrant number of chromosomes. This could happen either during oogenesis or spermatogenesis (aberrant partitioning of the chromosomes or chromatid during maternal or paternal meiosis, respectively) or, less frequently (about 3 %), during early division of the fertilized egg.

The occurrence of the maternal or paternal meiotic non-disjunction appears equally distributed in the KS patients (nearly 50 % each) [10]. In KS patients with an additional maternal X chromosome, non-disjunction in either the first or second meiotic division is most likely to have occurred, while in paternal cases the supernumerary X chromosome can only derive from a non-disjunction in the first meiotic division, since meiosis II error will result in either XX or YY gametes and therefore XXX or XYY zygotes [25].

The origin of the supernumerary X chromosome has also been associated with phenotypic differences, although evidence is not conclusive. In particular, it has been reported that KS patients with a paternal origin of the supernumerary X chromosome have a later onset and slower pubertal progression [26]. Other studies, however, suggested that the parental origin of the supernumerary X chromosome has no particular influence on the phenotype of the patients [27–29].

An advanced maternal, and possibly paternal, age has been reported as a risk factor for KS. A 4-fold increase in the prevalence of KS cases was showed in mothers aged >40 years, compared to mothers aged <24 [3]. The maternal age effect was also shown in KS patients with post-zygotic mitotic non-disjunction. Indeed, the first three mitotic divisions are controlled by maternal protein and RNA; thus, with the increase of the mother age, the chance of mitotic errors increases accordingly and the possibility of KS of post-zygotic origin as well. On the contrary, only some, albeit debatable, evidences for a relation with sex chromosomal trisomies and advanced paternal age were so far demonstrated [30].

Mosaicism (mainly 46,XY/47,XXY) is present in around 10–20 % of the KS patients and arises from either non-disjunction in an early mitotic division of the developing 46,XY zygote, or from loss of one of the X chromosome of a 47,XXY conception due to anaphase lagging.

### Peculiar genetic aspects in KS

#### X chromosome inactivation and gene dosage

In the somatic cells of females, the transcription of one of the two X chromosome is known to be randomly inactivated in order to ensure a dosage-compensation of the X-encoded genes to that of male cells. Although several genes are escaping inactivation, the Barr body (sex chromatin) in female cells is microscopically identifiable and represents the visible inactivated X chromosome [31]. The untranslated RNA product of the X-inactive-specific transcript (Xist) gene, located on the long arm of the inactive X chromosome, mediates the coating and silencing of the extra X chromosome in human somatic cells [32, 33]. Thus, the expression of Xist indicates the presence of the second and any other supernumerary X chromosome in the somatic cell [34]. Recent studies demonstrated that Xist methylation in KS patients and in the 41,XX^Y^ KS mouse animal model is comparable with the one observed in female subjects [35–37]. These data together with the expression of Xist in the blood cells of KS patients, while not in healthy 46,XY men [38] and the findings of the Barr body in KS Leydig and Sertoli cells [39, 40] probably means that the somatic cells in KS males inactivate properly the extra X chromosome as the female cells. Thus, any increase in gene dosage in these cell types will only concern genes that escape the X chromosome inactivation. Indeed, it is estimated that around 15 % of X-linked genes in humans and thirteen genes in mice escape transcription inactivation (possibly skewed) to some degree [41–46], and many more show a cell-type-specific inactivation pattern [44]. The genes that escape inactivation are mapping prevalently on the short arm of the X chromosome (Xp). Nevertheless, these genes that escape X inactivation are putatively contributing to the KS phenotype, since they would be present in double gene dosage in male patient KS, whose metabolism may not be suitable for female dosage of certain X-linked genes. Werler et al. [47] have demonstrated that 4 genes (Eif2s3x, Ddx3x, Kdm5c, Kdm6a) that escape the X inactivation either in human and mice, present different expression profile in different organs of the 41,XX^Y^ KS mouse model. They are equally or less expressed in the liver and kidney of, respectively, 40,XX or 40,XY mice, while they are more expressed in the brain of the 41,XX^Y^ mice compared to the normal karyotyped mice, either male or female.

Moreover, the skewed X chromosome inactivation, defined as the preferential inactivation of one of the two X chromosome in female, is present in the KS patients as well and this phenomenon may influence the clinical phenotype.

The situation in the germ cells seems to be different and more complex since the X inactivation in these cells follows a distinct pathway [48, 49]. Earlier studies demonstrated that germ cells were the only cell type in the testis expressing the Xist and this allows the first conclusion that the unique X chromosome in male germ cells was inactivated in the adult testis [50]. Nevertheless, later studies have shown that X inactivation does not fully occur in adult spermatogonia since a large number of X chromosome genes are expressed in the testicular germ cells [51]. Indeed, the complete sequencing of the human X chromosome showed that around 10 % of X-linked genes (99 genes) are testis specific and belong to the so-called cancer-testis antigens family [52]. It was demonstrated that X reactivation occurs during the germ cell development in the 41,XXY mouse model, and it is assumed that a proper X chromosome gene dosage is crucial for the survival of germ cell in the mature testis [53]. Thus, either in the germ cells of the KS patients, the altered X-linked gene dosage of these testis specific genes, due to their X inactivation escape, may compromise testicular function or influence the meiotic process itself and therefore play a role in the etiology of infertility in KS males [54–57]. A recent study demonstrated that the over expression in the mouse germ-cell-derived GC-1 and GC-2 cells of the gene Testis-expressed 11 (TEX11), an X chromosome-encoded germ-cell-specific protein that is expressed most abundantly in spermatogonia and early spermatocytes in the testes, results in a suppression of the cell proliferation [58]. These results suggest that increased expression of TEX11 in the germ cells of KS patients, following the X inactivation escape, may partially contribute to the germ cell death and make TEX11 a potential candidate gene responsible for the KS spermatogenetic failure.

#### The androgen receptor

The androgen receptor (AR) gene, which mapped to Xq11.2-12, is of physiological importance in the testis and may play a particular role in differences of the KS phenotype. The N-terminal domain of AR gene exon 1 contains a stretch of CAG repeats, which is highly polymorphic. The length of this stretch is inversely correlated with the receptor activity [59]. In KS patients, one of the two AR alleles is inactivated [12, 60, 61], theoretically with the same probability. Nevertheless, Suzuki et al. [61] reported a preferential inactivation of the longer allele, while Zitzmann and colleagues the opposite [12]. Moreover, the KS series patients characterized by Zitzmann et al. [12] with the preferential inactivation of the shorter allele, and thus characterized by longer CAG repeats in their AR gene, tend to be more severely affected than those with the shorter CAG stretch. This correlation was found regarding their social status, body height, bone density, testicular volume, presence of gynecomastia and response to androgen substitution [12]. Another study demonstrated that KS patients with longer CAG stretch present later onset and slower progression of puberty and slower testicular degeneration process [26]. More recently another study confirmed the association with the CAG repeats and the phenotypic variability of the KS patients (positive correlation of the length of CAG stretch with final height and span and negative with cholesterol and hematocrit level) without any significant evidence either of preferential inactivation of the shorter allele or the correlation between the skewed X inactivation and the clinical manifestation of the analyzed KS series [13].

However, other studies [62, 63] did not found evidence for a preferential inactivation of AR with shorter or longer CAG repeats, nor found associations with some clinical features (osteoporosis, artery diameter) and weighted CAG repeat length.

#### Activity of the genes located in the pseudoautosomal regions (PAR)

The pseudoautosomal regions (PAR1 and PAR2) are short homologous regions between the X and Y chromosomes in mammals. The PAR behave like an autosome and recombine during meiosis. Thus genes in this region are inherited in an autosomal rather than a strictly sex-linked manner [64]. PAR1 is located at the terminal region of the short arms and PAR2 at the tips of the long arms of these chromosomes [64]. To date, 24 genes have been assigned to the PAR1 region [52, 64], being half of them with a known function. PAR1 is required during male meiosis for X–Y chromosomes pairing, a process which is known to have a critical function in spermatogenesis, at least in humans and mouse [65–67]. In contrast, so far only 4 genes have been discovered in the PAR2 region [52, 64]. All characterized genes within PAR1 escape X inactivation, which means that it is normally present a double gene dosage of these gene product in males and female. Moreover, this also means that in KS male three active copies of the X–Y homologous genes of PAR will be present with possible influence in modulating the clinical phenotype. Of these genes, the only one that has been clearly shown to influence the phenotype in KS is the Short-stature Homeobox-containing gene on chromosome X (SHOX) situated in the PAR1 on Xp. As already mentioned above, in KS tall stature and long extremities are evident since the early childhood despite normal circulating levels of IGF-1 and IGFBP-3. This suggests that the sole hypogonadism cannot explain completely this phenotype and, indeed, the excessive expression of growth-related genes such as SHOX is implicated [68]. Moreover, brain natriuretic peptide and fibroblast growth factor receptor 3 are transcriptionally targets of SHOX [69, 70] and further studies on this molecular interaction may enhance our understanding of the phenotypic consequences of the syndrome.

#### Copy number variations in the X chromosome

Other than gene dosage effects and parental origin of the supernumerary X chromosome, recent evidence suggested that additional features of the X chromosome might have a role in phenotypic differences among KS subjects. In particular, it has been found that KS subjects have more frequently than controls X-linked copy number variations (CNVs) (41.5 vs 28.6 % of females and 18.6 % of males) [71]. The number of X-linked CNVs in KS patients was also higher with respect to that found in control females and males. Importantly, almost all of the X-linked CNVs in KS subjects were duplications, half of the X-linked CNVs fell within regions encompassing genes, and most of them (90 %) included genes escaping X inactivation in the regions of X–Y homology, particularly in PAR1 and Xq21.31. This means, for example, that duplication in these genes in KS subjects increases the copy number (and the expression) to four rather than to three as in KS men without a duplication, suggesting that X-linked CNVs (especially duplications) might contribute to the clinical phenotype [71].

## Hypogonadism and related phenotype

Hypogonadism remains silent until pubertal onset. Data on serum testosterone and estradiol in healthy prepubertal children are scanty, and there are no studies investigating sex steroids secretion in KS during infancy [24]. Usually, boys with KS enter puberty regularly and testosterone rises in a physiological way allowing epiphyseal closure and satisfactory development of secondary sexual characteristics (i.e., penile size, scrotum morphology and pubic hair distribution) [24, 72–75]. At puberty, only few patients develop overt hypogonadism, with evident signs (horizontal pubic line, scant body, axillary, and facial hair, poor muscle mass) and symptoms of under-virilization and/or delayed puberty [1, 5, 7, 10, 11]. Low to normal serum testosterone at puberty contributes in part to the development of tall stature and worsens the ratio between upper to lower skeletal segments by exacerbating the growth of legs that are still longer since infancy [17]. Some authors also hypothesized that androgen deficiency in the first trimester of life (during mini-puberty) might contribute to these skeletal features, but clear evidence is still lacking [17] (Table 1).

Serum T concentrations tend to fall to the mid-low range in the young adult with KS [22, 76] in accordance to the appearance and/or worsening of hypogonadal signs and symptoms (Fig. 2). However, the age of onset of hypogonadism is extensively variable [5]. In literature, lower than normal serum T concentrations (<12 nmol/L) is found in variable percentages (65–85 %) of adults with KS, although serum T can sometimes be in the normal range [5]. Hypogonadism is always coupled with elevated gonadotropins (hypergonadotropic hypogonadism) and the latter are usually higher than normal even in patients with serum testosterone still in the normal range [1, 5, 10, 77, 78]. Due to heterogeneous values of serum testosterone in KS, the adequate threshold below which serum T should be considered insufficient in these patients is lacking. Controlled studies showing a different age-related hypogonadism in patients with KS are not available, so the use of inter-society guideline criteria for male hypogonadism seems to be, at present, the most appropriate one also in this context [79, 80].

Other reproductive hormones might be altered in KS. Serum estradiol might be almost normal or sometimes elevated, but the estradiol to testosterone ratio seems to be constantly higher than in normal men [5, 81] (Table 1). This may account for the development of gynecomastia, the latter being associated with low testosterone even in non Klinefelter patients [82]. In adult men with KS, serum inhibin B is undetectable due to the tubular damage [83, 84] and serum anti-mullerian hormone is lower than normal [76, 85–87]. More recent studies have provided also evidence of lower INSL3 levels in comparison with normal subjects [88].

Likewise signs and symptoms of hypogonadism (e.g. sexual dysfunction), comorbidities associated with KS such as diabetes, metabolic syndrome, osteoporosis and cardiovascular diseases usually appear during adulthood and increase with advancing age [16, 22, 89].

When serum testosterone is below normal, obesity and gynoid fat distribution are common in men with KS [16], in addition reduced muscle strength may develop [5, 7, 10, 11].

Testosterone replacement therapy is effective in improving symptoms related to androgen deficiency, but not all other features related to the genetic abnormality (Table 1). For this reason, it is important to unravel symptoms due to testosterone deficiency from the others.

Several other clinical features of KS have been related to hypogonadism, but with a variable degree of uncertainty. The finding of bone mineral density lower than normal is prevalent in patients with KS, but it seems not directly related to low serum testosterone [90]. Accordingly, several cognitive and psychological aspects are associated with KS [5]. Intellectual abilities are not impaired, but deficits in specific domains of cognition (e.g. reduction in speech and in language abilities, verbal processing speed) may be present [19, 20, 91] and the school performance may be impaired. Speech disabilities remain confined in the range of normal general cognitive abilities [92]. The overall cognitive ability standard score, in fact, on average falls within the normal range and not in the intellectual disability range [92, 93]. As language and learning disabilities become manifest during infancy, their relationship with hypogonadism may be ruled out. In addition, this kind of speech problems is common also in other sex chromosome trisomy not associated with hypogonadism [93], thus suggesting that they depend from genetic factors [92]. However, it is not possible to exclude that early exposure to low androgens levels during prenatal life might represent a causal factor for the development of speech disabilities. Several psychiatric disorders (e.g. depression, paraphilia, autistic and obsession-compulsive trait) seem to be more common in KS, but these data need to be replicated on a large scale in order to be confirmed [93, 94], their relationship with hypogonadism remaining unknown [92]. Finally, the old concept of a strong association among KS and criminal behavior, severe psychiatric disorders, and mental retardation is now considered outdated, since no evidence-based data have subsequently confirmed this erroneous long-held view [5, 7, 10, 11].

A case of a 51-year-old adult man affected by both KS and congenital adrenal hyperplasia (CAH) due to 21 hydroxylase deficiency, the first causing androgen deficiency, the latter leading to androgen excess was helpful in disclosing testosterone-dependent signs and symptoms in KS [95]. Under-virilization and abnormalities of sexual behavior (in particular of libido, erectile function and sexual intercourses) occurred in this patient soon after starting cortisone acetate, due to the reduction in adrenal steroids and the impairment of the balance in the androgen status previously created by the two syndromes [95]. Thus, the normalization of adrenal androgens revealed clinical features due to testosterone deficiency and KS [95].

## Clinical implications

Patients with a diagnosis of KS needs to be followed throughout life and have to be treated with testosterone in case of hypogonadism. Particular attention should be paid to adequate titration of testosterone dosage in these patients since they mostly have a mild testosterone deficiency, especially those with mild phenotype. All testosterone formulations are effective in patients with KS, the choice depending on the pretreatment levels of serum T, patients’ preferences and physicians’ attitude and experience with the formulations commercially available.

Finally, the management of KS should also include the prevention or treatment of comorbidities.

## Unresolved issues

The main issue concerns how to improve a precocious diagnosis in order to reduce the number of patients who remains undiagnosed and to avoid the delay in the diagnosis. For these patients who are unaware of suffering of KS, it could be assumed that they are somewhat healthy and do not require medicalization and/or treatment. However, we do not know how many of them do not seek medical consultation for other reasons (lack of compliance, negligence, scarce attitude to consult physician, etc.), but still complain of signs and symptoms of the disease. This gap in knowledge does not allow unraveling the entire spectrum of phenotypes of KS, a prerequisite useful to target and personalize the management of the disease, according with the real patient’s health status.

In addition, some genetic aspects related to the phenotype remains undetermined. Despite the insights provided by numerous studies concerning the clinical consequences of KS performed so far, our knowledge of the molecular and cellular mechanism underlying the KS pathogenesis is still limited in part due to the lack of in-depth mechanistic studies. A part from the aneuploidy per se and the inter-individual genetic variation, several genetic mechanisms may play as other possible modulators of the variability of the phenotype observed in KS patients. It deals with the dosage effect and the expression/inactivation status of the X chromosome genes, the presence of mosaicism, the number and the derivation (maternal or paternal) of supernumerary X chromosome/s, the activity of the genes located in the pseudoautosomal regions (PAR) of the sex chromosomes.

Furthermore, we do not know, at present, how to manage paucisymptomatic KS patients that remains undiagnosed since our knowledge on their real health conditions is very poor.

Hence, the impact of the disease during fetal life and the early period after birth (including the so-called minipuberty) remains an open issue. Furthermore, data on how patients with KS aging are scanty as well as information on how managing these patients in the elderly.

## Conclusions

Clinical and genetic phenotype of KS as well as their relationship are still not completely understood and need to be fully elucidated in order to improve also the clinical management of this disease.

Accordingly, KS remains largely underdiagnosed (only 25 % of the expected number of patients are correctly diagnosed and only a minority before the puberty onset) and the majority of patients are often diagnosed during adulthood. Prompt educational and psychological supports might prevent any difficulties in their language, scholastic and neuropsychological difficulties; the start of the testosterone replacement therapy as soon as the patients need it allows them to avoid the long-term consequences of the hypogonadism; semen or testicular tissue cryopreservation could also be performed as soon as possible, before the testicular damage starts, probably at puberty. Indeed, a major effort should be done in order to increase our ability to perform early diagnosis of the KS and to provide advancement on the knowledge of both pathogenetic and clinical issues.
